# Physiological tests for yeast brewery cells immobilized on modified chamotte carrier

**DOI:** 10.1007/s10482-013-9978-1

**Published:** 2013-07-26

**Authors:** Joanna Berlowska, Dorota Kregiel, Wojciech Ambroziak

**Affiliations:** Institute of Fermentation Technology and Microbiology, Technical University of Lodz, ul. Wolczanska 171/173, 90-924 Lodz, Poland

**Keywords:** Yeast immobilization, Chamotte, SDH activity, PDC activity, In situ assay

## Abstract

In this study yeast cell physiological activity was assessed on the basis of the in situ activity of two important enzymes, succinate dehydrogenase and pyruvate decarboxylase. FUN1 dye bioconversion and cellular ATP content were also taken as important indicators of yeast cell activity. The study was conducted on six brewing yeast strains, which were either free cells or immobilized on a chamotte carrier. The experimental data obtained indicate clearly that, in most cases, the immobilized cells showed lower enzyme activity than free cells from analogous cultures. Pyruvate decarboxylase activity in immobilized cells was higher than in planktonic cell populations only in the case of the *Saccharomyces pastorianus* 680 strain. However, in a comparative assessment of the fermentation process, conducted with the use of free and immobilized cells, much more favorable dynamics and carbon dioxide productivity were observed in immobilized cells, especially in the case of brewing lager yeast strains. This may explain the higher total cell density per volume unit of the fermented medium and the improved resistance of immobilized cells to environmental changes.

## Introduction

Current research on the application of immobilized yeast cells in brewing technology has three main focuses: the production of alcohol-free beer, and conducting either main fermentation or green beer maturation in continuous systems. Only a few of the technologies proposed in the literature have resulted in pilot-scale attempts or industrial implementation (Verbelen et al. 2006; Willaert 2000; Brányik et al. 2012). One of the main difficulties is maintaining the desired physiological state in immobilized microbial cells. The outcome of a brewery fermentation depends on wort composition, ambient technological conditions as well as on variations in pitching yeast activity. Therefore, for the end product quality, monitoring of yeast physiological state is very essential.

The term “physiological activity” could describe various important parameters: fermentation potential, stress tolerance, aging, growth or reproduction abilities. The physiology of immobilized cells is affected by the microenvironment and the supply of nutrients and metabolic products (internal and external mass transfer) (Junter et al. 2002; Pajić-Lijakovic et al. 2007; Gonga et al. 2010). Cellular stress at the stage of immobilization may also have a significant impact on the physiological state, and according to Smart (2001) this physiological history may determine a cell’s efficiency during the technological process. The general aim is to maintain the greatest viability and metabolic activity of the cells, allowing to the process to be carried out with high efficiency for the longest time possible. Continuous system technologies in beer production require immobilized yeast cells to be kept for several months in bioreactors. In the case of yeast plankton populations, over time the linear dimensions of cells increase, there is a longer generation time and their metabolic activity decreases.

It has been shown that free and immobilized yeast cells differ in chemical composition and ploidy (Verbelen et al. 2006). Immobilized cells, in comparison with free cells, have a higher content of glycogen, trehalose, structural polysaccharides (glucans and mannan), fatty acids and DNA. Immobilization also causes changes in the proteome of a cell, in the level of gene expression, and has a significant impact on the quantitative composition and organization of the cytoplasmic membrane and cell wall structures (Brányik et al. 2008; Parascandola et al. 1997). Many studies have reported an increase in metabolic activity (increased rate of sugar uptake and productivity of selected metabolites) in immobilized cells (Junter et al. 2002; Norton and D’Amore 1994; Angelova et al. 2000; Talebnia and Taherzadeh 2007; Plessas et al. 2007; Li et al. 2007; Behera et al. 2011). Adsorption of *Saccharomyces carlsbergensis* on porous glass and *S. cerevisiae* on ceramic support resulted in increased production of ethanol and reduced production of CO_2_ (Kourkoutas et al. 2004). In yeast entrapped in alginate matrices, a slight decrease was noticed in intracellular pH due to increased enzymatic activity. This promotes the permeability of membranes, which in turn leads to an increase in proton transport and ATP use, stimulating glycolysis processes (Galazzo and Bailey 1990). Higher efficiency in the pentose phosphate pathway and of glycolytic flux may also be explained by the increased activity of alcohol dehydrogenase and by more efficient regeneration of the NADH and NADPH cofactors Brányik et al. (2008).

Many changes have been made in the modern beer-brewing process since the first recorded beer production by mankind. However, despite all these changes, one constant factor is the requirement for good quality brewing yeast (Lodolo et al. 2008). Knowing the physiological state of immobilized yeast cells is important not only from a theoretical perspective. It is important to verify the efficacy of cell-carrier systems, as well as to monitor the continuous process. The precise evaluation of yeast physiology is rather difficult and sometimes problematic—the type of information gathered depends on the kind of analytical method applied. Therefore, monitoring of yeast physiology should be multi-parametric.

Observation, analytics and diagnostics of biofilms formed on abiotic surfaces are usually complicated, and often expensive. Visualization of the spaces colonized by the microorganisms and the architecture of the three-dimensional structure formed is made possible by such techniques as magnetic resonance imaging, optical coherence tomography, confocal laser scanning microscopy and fluorescence microscopy (Chandra et al. 2001; Nott et al. 2005; Xi et al. 2006). The coupling of fluorescent in situ hybridization (FISH) and microautoradiography allows for the consumption by the tested microorganisms of different substrates to be determined precisely (Lee et al. 1999; Kindaichi et al. 2004). In the description of the interactions between immobilized microorganisms and their metabolic characteristics, the use of microelectrodes and a combination of FISH, mass spectroscopy and isotopic labeling techniques can be of significant help (Jang et al. 2003; Majors et al. 2005). Many techniques used in the evaluation of the microbial physiology of immobilized cells require their detachment (Uppuluri et al. 2006). Multiple cell rinsing and centrifugation can have a significant impact on the value of the parameter under evaluation (Brányik et al. 2005). In the current study, we propose determining succinate dehydrogenase (SDH) and pyruvate decarboxylase (PDC) activity in situ in immobilized cells with increased membrane permeability. The aim of noninvasive analysis (in situ) is to avoid disturbing the normal functioning of the cells and so lowering their physiological activity. An in situ enzyme activity assay based on chemical changes in membrane permeability allowing migration of low molecular weight compounds (substrates, products, cofactors), while the enzymes and other macromolecules are kept in constant concentrations (Freire et al. 1998). The cytoplasmic membrane forms a barrier with low permeability and enzyme activity is determined in whole cells (Cordeiro and Freire 1995; Kippert 1995; Bindu et al. 1998; Kondo et al. 2008; Crotti et al. 2001; Gough et al. 2001; Chelico and Khachatourians 2003; Berlowska et al. 2006, 2009; Miranda and Ferreira 2008).The advantage is that enzyme activity is determined for a specific physiological state of the cell. Using this type of enzyme assay, cellular regulatory effects can be observed and enzyme activities determined for cells immobilized on solid supports.

We focused our research on the immobilization of yeast brewery strains on chamotte ceramic carriers. Our study is the continuation of previous research on yeast adhesion to native and modified chamotte tablets (Kregiel et al. 2012, Berlowska et al. 2013), which led us to study chamotte modification as a way to enhance yeast cell adhesion efficiency. Enhanced in this way yeast immobilization and proper selected conditions of this process give opportunity to conduct physiological tests for adhered cells.

The aim of the present study was to determine the effect of the immobilization process on brewing yeast cell physiological activity. Multi-parameter physiological activity evaluation was conducted for free and immobilized yeast cells, which allowed the nature, characteristics, fermentation abilities and technological suitability of the tested strains to be described. The basis of physiological activity assessment was the activity assay in situ of two enzymes, SDH and PDC, important yeast metabolic pathways (the Krebs cycle and glycolysis respectively). PDC (EC 4.1.1.1) is an important enzyme for yeast fermentation. Measuring PDC activity allows the fermentation activity of individual yeast strains to be monitored. On the other hand, SDH (EC 1.3.5.1) is essential for the aerobic utilization of carbon sources and plays a crucial role in the supply of energy for the physiological activity of yeast cells (Kregiel et al. 2013). Therefore, SDH and PDC activity assays may be the important methods to evaluate yeast activity and control different biotechnological processes. ATP content and fermentative activity were also monitored.

This paper is the first to describe the physiological activity of brewery yeasts immobilized on inexpensive, porous chamotte covered by active organo-silanes.

## Materials and methods

### Carriers

During the experiment, solid carriers were used made from inexpensive chamotte material [mainly Al_2_O_3_ (36 %) with SiO_2_ (58 %) and Fe_2_O_3_ (2.6 %)]. The ceramic chamotte tablets were prepared from water and chamotte fire clay (50–1,000 μm) (Boleslawiec Refractory Plant BZMO Ltd., Poland) in the ratio 1:2. Chamotte carriers were made in our laboratory by firing chamotte fire clay at a temperature of 1,100 °C. The height and diameter of the chamotte tablets were 5 and 15 mm, respectively.

The carriers were modified in the Centre of Molecular and Macromolecular Studies of the Polish Academy of Science. The chamotte tablets were immersed in 10 % H_3_PO_4_, left for 2 h and then washed and dried at room temperature. The dry tablets were placed in 10 % 3-(*N*,*N*-dimethyl-*N*-2-hydroxy-ethyl) ammonium propyldimethoxysilane or (3-glycidoxy propyl) trimethoxysilane chloride isopropanol solution. The flask was repeatedly connected and disconnected from the vacuum pump to remove the air from and draw the silane solution into the pores. The prepared media were pre-dried to evaporate the isopropanol and then incubated at 80 °C for 12 h. For the (3-glycidoxy propyl) trimethoxysilane modification, dry carriers were laid on a Buchner funnel so as to form a single layer and their location was changed periodically (by turning them upside down). Under a funnel, 200 mL of 25 % ammonia-water was heated and stirred for 2 h. Free ammonia caused the collapse of the epoxide ring on the surface and creation of 3-(3-amino-2-hydroxy-1-propoxy) propyldimethoxysilane groups.

### Yeast strains

In our research, to assess physiological state of immobilized brewery yeasts, the six various top and bottom-fermenting *Saccharomyces* stains were used (Table 1.) These strains were selected according to their adhesion properties in the previous studies (Kregiel et al. 2012; Berlowska et al. 2013).

**Table 1:** Applied biological material and immobilization methods

Strain	Type	Collection	Immobilization medium
*S. pastorianus* W 34/70	Lager	Hafebank Weihenstephan (DE)	Ringer’s solution
*S. pastorianus* 680		National Collection of Yeast Cultures (GB)	Wort broth
*S. pastorianus* B4		Collection LOCK105 (PL)	Wort broth
*S. cerevisae* TT	Ale		Ringer’s solution
*S. cerevisae* 1017		National Collection of Yeast Cultures (GB)	Wort broth
*S. cerevisae* 1183			Ringer’s solution

The yeast strains were stored on wort agar slants (Merck) at room temperature. Directly prior to the experiment, they were activated by placing them on fresh agar slants and incubated at 30 °C for 48 h.

### Culture conditions

The yeasts were propagated in liquid wort broth (Merck). The cultivation was carried out in a 500 mL round bottom flask filled with 50 mL of medium with 1 % v/v yeast suspension added. It was performed using the shaking flask method (220 rpm) at a temperature of 30 °C.

For the purpose of the experiment, starved cultures were also required. These were prepared from stationary phase cultures which were rinsed twice with Ringer’s solution (Merck) and resuspended in the same solution.

### Fermentations

To assess fermentation performance on a small laboratory scale, for consistent and reproducible procedures the simple, well defined glucose-based medium was used. Static fermentations were conducted in 50 mL medium ([(NH_4_)_2_·SO_4_ 3 g/L; KH_2_PO_4_ 1 g/L; MgSO_4_·7H_2_O 0.5 g/L; yeast extract (Difco) 0.5 g/L; CaCO_3_ 3 g/L] with 12 % glucose) sealed with a fermentation lock containing paraffin oil. The fermentations, carried out both for adhered and free yeast cells, were conducted over 7 days at the appropriate temperature (top fermenting yeast 20 °C, bottom fermenting yeast 10 °C).

### Yeast cell immobilization

Only the high efficiency of immobilization let us to evaluate physiological state of adhered yeast cells. Therefore, the parameters of adhesion process have been optimized for each strain on the base of previous studies results. Both character of chemical surface modifications and immobilization medium were taken into consideration (Kregiel et al. 2012; Berlowska et al. 2013). The yeast cells were harvested when they reached the appropriate phase of growth or physiological state, at which point they were standardized. In 50-mL sterile Erlenmeyer flasks, 5 mL of cells and medium suspensions with a density of 5 × 10^7^ cells/mL were prepared. For dilutions, sterile basic cultivation (wort broth) medium or Ringer solution was used (Table 1). Next, sterile carriers were introduced into each of the previously prepared suspensions and incubated at 30 °C with agitation (75 rpm) for 24 h.

### Determination of the number of immobilized yeast cells

After the adhesion process, five pieces of chamotte tablet were selected from each experimental sample. The tablets were suspended in 5 mL of 5 % H_2_SO_4_. Then, tubes filled with tablets suspended in appropriate solutions were boiled for 2 min. and vortexed for 15 min, after which the carriers were removed. The remaining solution was analysed for the number of microorganisms. The density of the yeast suspensions was determined using the fluorimetric method based on DAPI staining (Kregiel et al. 2012).

### Succinate dehydrogenase (SDH) activity

The in situ assay measured SDH activity in whole cells. After pre-incubation with digitonin, a permeabilization agent blue tetrazolium salt (BT), in the presence of phenazine methosulfate and sodium azide, was reduced intracellularly to colored formazan crystals. The amount of the formed formazan was determined spectrophotometrically after DMSO extraction (Kręgiel et al. 2008). Free cells were measured in standardized suspensions (3 × 10^8^ cells/mL). In the case of the immobilized yeasts, 6 chamotte carriers with adhered cells were used (no centrifugation for cells separation was required). Knowing the number of immobilized yeast cells, the SDH activity values were recalculated appropriately.

### ATP content

Intracellular ATP content was determined in relative light units (RLU) on the basis of the luciferin/luciferase method using a Hy-Lite2 luminometer (Merck) (41). The measurement of free cells was conducted in standardized suspensions (1 × 10^4^ cells/mL). Tablets with immobilized cells were washed with sterile distilled water, and 1 mL of Somatic Cell ATP Releasing Reagent (SIGMA-ALDRICH) was added to each carrier. After 5 min, the solution with released ATP was diluted to an equivalent concentration of 1 × 10^4^ cells/mL (having determined the number of immobilized yeast cells) and analyzed. The readings in RLU were calculated on the basis of the standard curve and expressed in fg/dm^3^ of ATP.

### Pyruvate decarboxylase (PDC) activity

PDC activity was measured in situ in whole cells with digitonin permeabilized membranes. Sodium pyruvate (0.05 M) solution was used as a reaction substrate. The acetaldehyde formed was detected using the GC technique with a Headspace Autosampler (Berlowska et al. 2009). Measurements of the free cells were conducted in standardized suspensions (2 × 10^8^ cells/mL) and in the case of immobilized cells using four carriers (no centrifugation for cell separation was required). Once the number of immobilized yeast cells had been determined, the PDC activity values obtained were recalculated appropriately.

### Fermentation activity

The fermentation activity of yeast populations was evaluated by a quantitative determination of the carbon dioxide production in grams per 100 mL of fermentative medium.

### FUN1 staining

Tablets with immobilized cells were rinsed with distilled water to wash out the medium that remained on the surface. To stain the cells with FUN1 they were first soaked in a solution of 2 % glucose in 10 mM Na-HEPES. Then, 200 μL of 0.1 μg/mL FUN1 was poured onto the surface of each tablet. After 5 min incubation at 30 °C, the tablets were left to dry and then examined under a fluorescence microscope OLYMPUS BX 41 equipped with the appropriate filter (excitation wavelength 470–490 nm).

### Statistical method

Each experiment was performed in triplicate and each datum was the arithmetic mean (a.m.) of three measurements. The standard deviations (SD) were calculated and the results given as am ± SD.

## Results and discussion

### Yeast immobilization

**Fig. 1:**
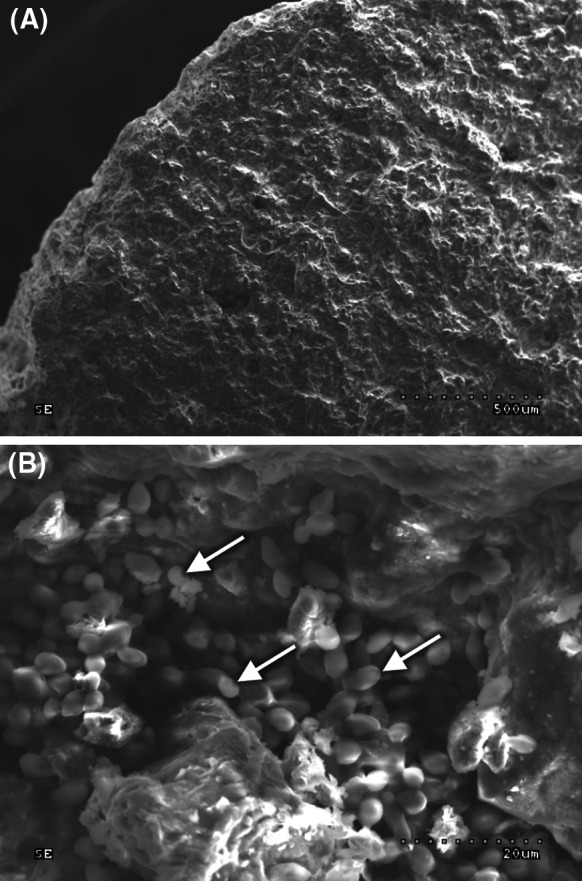
Chamotte surface: **a** native, **b** with immobilized cells

For the purposes of the present study, six brewing yeast strains were immobilized on chamotte carriers with chemically modified surfaces (Fig. 1a). The number of cells per carrier was assayed fluorometrically using DAPI. This cationic dye specifically binds to DNA in places rich in adenine–thymine pairs. It is also accumulated in small grooves of the DNA double helix (Barker and Smart 1996). According to the authors’ own research, the amount of emitted light, measured spectrofluorimetrically, is proportional to the number of stained, heat denatured, yeast cells. The effectiveness of the adhesion processes ranged from 2.6 to 4.0 × 10^7^ cells per cm^2^. The spatial distribution of immobilized microorganisms was imaged using a scanning electron microscope HITACHI S-3000N (Fig. 1b).

### SDH activity

Evaluating the activities of dehydrogenases is used more and more commonly as a method of determining the physiological state of microbial cells. Water-soluble, colorless tetrazolium salts are reduced to color formazans by dehydrogenases coupled with the electron transport system. SDH (EC 1.3.99.1), integrally connected to the inner mitochondrial membrane, catalyzes the dehydrogenation of succinate to fumarate. Cells that reduce tetrazolium dyes are treated as alive, and while those that do not reduce these salts cannot unambiguously be considered dead, they have lost their respiratory function (Breeuwer and Abee 2000; De Nooijer et al. 2005).

The highest relative SDH activity of immobilized cells was estimated for *S. pastorianus* W 34/70, and *S. cerevisiae* 1017. Higher enzyme activity after cell adhesion in comparison to the cells from young free aerobic cultures was observed only in the case of *S. pastorianus* W 34/70. For the remaining five microorganisms, a significant decrease in SDH activity was associated with cell adsorption on solid surfaces. These proportions were changed during the 7-day incubation period in the medium in which the process of immobilization was carried out. Aging of immobilized microorganisms suspended in wort broth resulted in higher levels of enzyme activity than in the case of free cells (Table 2).

**Table 2:** SDH activity of free and immobilized yeast cells

Strain	SDH activity (amount of μmol of formazan/3 × 10^8^ cells)			
	Cells from stationary phase	Cells after adhesion	Free cells after 7-day incubation	Immobilized cells after 7-day incubation
(A) Adhesion in wort broth				
*S. cerevisiae* 1017	0.81 (±0.09)	0.15 (±0.02)	0.03 (±0.01)	0.08 (±0.01)
*S. pastorianus* 680	0.82 (±0.08)	0.02 (±0.00)	0.03 (±0.01)	0.08 (±0.01)
*S. pastorianus* B4	0.04 (±0.01)	0.02 (±0.00)	0.03 (±0.01)	0.05 (±0.01)
(B) Adhesion in Ringer’s solution				
*S. cerevisiae* TT	0.13 (±0.02)	0.04 (±0.01)	0.04 (±0.01)	0.03 (±0.01)
*S. cerevisiae* 1183	0.33 (±0.05)	0.07 (±0.02)	0.06 (±0.01)	0.03 (±0.01)
*S. pastorianus* W 34/70	0.24 (±0.02)	0.28 (±0.03)	0.04 (±0.01)	0.03 (±0.01)

Similar phenomena were described by Tsukatani et al. (2003) in studies of yeast vitality conducted with WST-1 tetrazolium salt. The amount of formazan reached its maximum value in the stationary phase of growth and fell to a quarter on the sixth day of the culture.

For the immobilized microorganisms suspended in Ringer’s solution, lower or similar activity was observed than in the case of free cells (Table 2). These differences can be explained by the stronger nutrient deficit. For the same reasons, within this group of strains, a decrease in SDH was observed after 7 days with regard to the values noted after adhesion. The activity determined after a 7-day aerobic incubation of *S. pastorianus* 680 and B4 was higher than it was at the beginning of the process. This fact may be associated with the specific growth and aging patterns of brewing lager yeast strains.

### ATP content

It is well known that with the abandonment of adenosine triphosphate synthesis, the existing ATP rapidly degrades. This feature makes ATP a good marker of cell physiological activity (Imai and Ohno 1995; Sato et al. 2008; Guillou et al. 2003; Osorio et al. 2004). For the purpose of this study, a common method based on the luciferin/luciferase system was also adopted. This widely used procedure of ATP determination in solutions or cell suspensions was modified to measure the level of this nucleotide in yeast cells immobilized on solid supports through the use of a ‘somatic cell ATP-releasing reagent’.

For all the tested strains, similar relationships were observed between the ATP content of the free and immobilized cells. However, a substantial decrease in ATP concentration throughout the adhesion process, and after longer incubation under aerobic conditions, was noted.

It is worth of noting that ATP values determined from the free cells in the stationary phase were even several times greater than for those from the immobilized cells (Table 3).

**Table 3:** ATP concentration in free and immobilized yeast cells

Strain	ATP (fg/cell)			
	Cells from stationary phase	Cells after adhesion	Free cells after 7-day incubation	Immobilized cells after 7-day incubation
(A) Adhesion in wort broth				
*S. cerevisiae* 1017	168.89 (±2.75)	13.44 (±1.14)	21.81 (±2.22)	0.00 (±0.00)
*S. pastorianus* 680	24.18 (±0.21)	7.86 (±0.68)	0.00 (±0.00)	0.00 (±0.00)
*S. pastorianus* B4	49.22 (±3.98)	8.94 (±0.97)	2.75 (±0.33)	0.00 (±0.00)
(B) Adhesion in Ringer’s solution				
*S. cerevisiae* TT	58.27 (±5.21)	9.19 (±1.09)	1.57 (±0.22)	0.00 (±0.00)
*S. cerevisiae* 1183	51.97 (±4.43)	6.34 (±0.77)	3.98 (±0.51)	0.00 (±0.00)
*S. pastorianus* W 34/70	32.91 (±2.89)	3.99 (±0.45)	11.36 (±1.15)	0.00 (±0.00)

At the cellular level, ATP depletion is the earliest cell-damaging factor. In vivo, severe depletion of ATP leads to dysfunction, destabilization, and aggregation of many cellular proteins, including enzymes (Kabakov et al. 2002). Sustained lack of ATP is obviously lethal for the cell. On the contrary, a transient (reversible) drop in cellular ATP confers tolerance to the next energy-depriving exposure. Therefore, we can assume that very low ATP level in yeast cells after immobilization may be a result of cell adaptation mechanisms to different environmental conditions.

### PDC activity and fermentation activity

Pyruvate decarboxylase (EC 4.1.1.1) is one of the key enzymes in anaerobic yeast metabolism, so determining its activity allows one to describe the physiological state of cells and their fermentation abilities. It is a homotetrameric enzyme (EC 4.1.1.1) that catalyses the decarboxylation of pyruvic acid into acetaldehyde and carbon dioxide in the cytoplasm (Pronk et al. 1996). PDC activity was determined in cells cultured under aerobic and oxygen-limited conditions. Specific competition occurring between PDC and pyruvate dehydrogenase complexes explains the simultaneous coexistence of two metabolic pathways (glycolysis and the Krebs cycle) and aerobic alcoholic fermentation (Van Hoek et al. 1998; Flikweert et al. 1999). The highest PDC activity of immobilized cells incubated under aerobic conditions was observed in the *S. pastorianus* B4 strain. Slightly lower values were recorded for *S. cerevisiae* 1017 and *S. pastorianus* W 34/70. Except for *S. pastorianus* B4, the activity of the newly immobilized microorganisms was reduced considerably. In four out of the six examined strains (excluding *S. cerevisiae* 1017 and 1183), there was a further decrease in PDC activity after a 7-day incubation period. Nevertheless, comparing these values to the activity of 7-day populations of free cells, higher or comparable activity was observed in the cases of all immobilized cell strains adhered in wort broth and of the *S. pastorianus* W 34/70 strain (Table 4).

**Table 4:** PDC activity of free and immobilized yeast cells—aerobic cultivation

Strain	PDC activity (amount of μmol of acetaldehyde/1 × 10^8^ cells)			
	Cells from stationary phase	Cells after adhesion	Free cells after 7-day incubation	Immobilized cells after 7-day incubation
(A) Adhesion in wort broth				
*S. cerevisiae* 1017	4.50 (±0.39)	0.15 (±0.02)	0.53 (±0.04)	0.59 (±0.07)
*S. pastorianus* 680	1.60 (±0.09)	0.29 (±0.03)	0.04 (±0.01)	0.06 (±0.01)
*S. pastorianus* B4	4.06 (±0.51)	1.54 (±0.01)	0.02 (±0.00)	1.18 (±0.12)
(B) Adhesion in Ringer’s solution				
*S. cerevisiae* TT	2.63 (±0.25)	0.04 (±0.01)	2.78 (±0.03)	0.04 (±0.01)
*S. cerevisiae* 1183	2.58 (±0.35)	0.01 (±0.00)	0.87 (±0.09)	0.23 (±0.03)
*S. pastorianus* W 34/70	4.72 (±0.55)	0.38 (±0.04)	0.00 (±0.00)	0.29 (±0.02)

The conditions of 7-day aerobic shaking cultures, associated with oxidative stress and cell aging, caused a decrease in the enzyme activity and ATP content to almost trace values. The smallest reduction in metabolic activity was observed for the top fermenting yeast. The foam that can be observed forming on the surface of the fermented broth with greater oxygenation can therefore be associated with a higher resistance to oxidative stress factors.

**Fig. 2:**
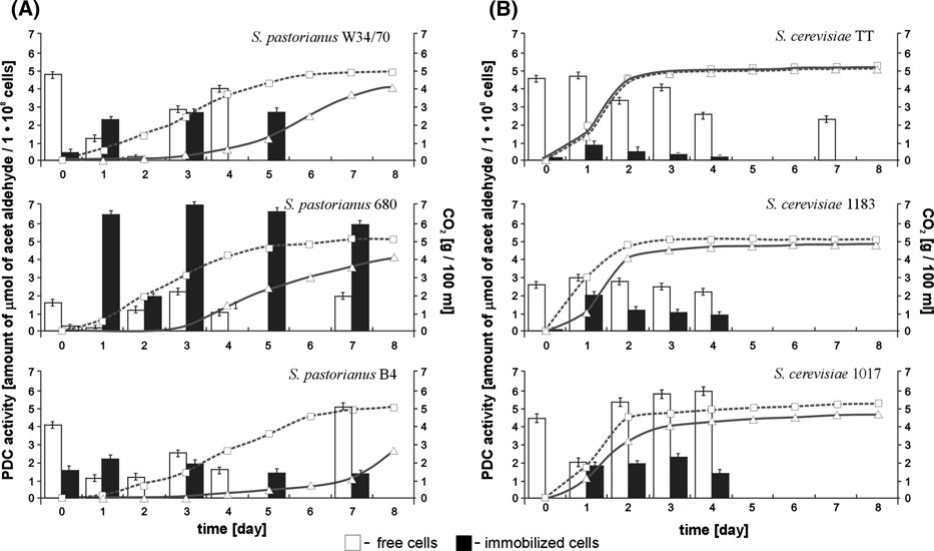
PDC activity of free and immobilized yeast cells—fermentation process. **a** Lager strains, **b** ale strains

Directly after adhesion, the carriers with immobilized cells were transferred to the fermentation medium. In parallel experiments conducted with free cells, changes in the composition of the medium and oxygen limitation caused a reduction in PDC activity (Fig. 2). On the other hand, we observed increased enzyme activity in the case of immobilized cells. The immobilization techniques also had no influence on the fermentation process. These phenomena can be explained by the increased stability and resistance to environmental changes of the immobilized yeast strains.

As in the previous tests, the PDC activity of immobilized microorganisms was lower in the case of most strains compared to the activity of planktonic populations. The exception was the immobilized cells of the *S. pastorianus* 680 strain, whose PDC activity was several times higher than in a free state. There is no known reason for this, but it could be due to an individual characteristic of the strain.

During the fermentation processes, the production of carbon dioxide was also measured. Changes in its values were similar for all *S. cerevisiae* strains (Fig. 2). Different fermentation dynamics were observed in the case of brewing lager yeast strains (Fig. 2a). These results suggest that high concentrations of immobilized cells per volume unit and their resistance to stress factors, including low temperature, could be responsible for a significant reduction in the adaptive phase and faster attenuation. Fermentation trials with immobilized cells showed that the immobilized yeasts adapted to the specific conditions. Despite the relatively low PDC activity of immobilized cells, the final fermentation effect, measured as the amount of CO_2_ produced, was achieved in a shorter time. Numerous examples of reduced fermentation times achieved both in continuous technologies using yeast cells adsorbed on inorganic carriers and in batch processes carried out with cells immobilized on natural supports have also been cited by Kourkoutas et al. (2004).

### FUN1 staining

**Fig. 3:**
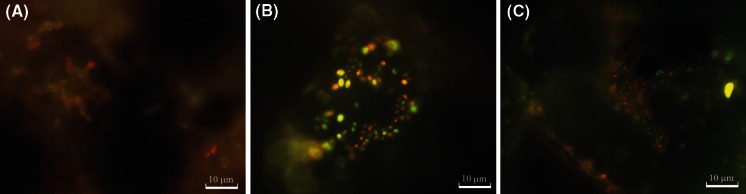
FUN 1 staining. **a** Stationary phase, **b** after adhesion, **c** 7-day fermentation

The metabolic activity (defined as the ability to fluorochrome bioconversion) of yeast strains was made visible using FUN1 stain. An enzyme activity assay and ATP determination of immobilized cells were conducted using more than one carrier. So the average value characterized the whole population. A large number of yeast cells with low enzyme activity were capable of bioconverting FUN1. Therefore, fluorescence staining revealed the diversity of metabolic activity in immobilized yeast cells (Fig. 3). This fact suggests an absence of physiological homogeneity in these populations.

### Physiology of immobilized yeast cells

Immobilization is ranked as one of the factors protecting yeast cells from adverse environmental effects (Junter et al. 2002; Norton et al. 1995; Kanda et al. 1998; Jirku 1999; Qun et al. 2002). In fermentations carried out with immobilized yeast cells, chemical changes in cellular composition were associated with an increased resistance to stress—especially to high concentrations of ethanol, and to the high gravity of the fermented wort (Verbelen et al. 2006; Brányik et al. 2008; Kourkoutas et al. 2004). Yeast cells immobilized in different polymer matrices were characterized by increased stability during freezing and freeze-drying. Osmotic stress also caused the production of polyols, and consequently an increase in resistance to toxic substances (Kourkoutas et al. 2004).

In our research, osmotic stress (after a change of wort broth or Ringer’s solution for the fermentation medium) and limited oxygen conditions did not cause a deterioration of metabolic activity in the immobilized yeast cells tested.

The differences in the activity of key enzymes assayed on free and immobilized cells in the seventh day of aerobic culture provide evidence of different aging processes. Comparing the age that brewer’s yeast reaches in traditional technologies with several months of continuous processes, the changes in the physiology of immobilized cells seem to be significant. A decrease in the viability and physiological activity of immobilized industrial yeast cells was also reported during the technological process (Calinescu et al. 2012). Both the aging of immobilized microorganisms and its impact on the quality of the resulting product are still unsolved issues (Brányik et al. 2005).

Metabolic changes caused by the immobilization process have been reported by many authors. An example is the increase in the amount of diacetyl in young beer obtained using immobilized yeast cells. This may be due to the inhibition of biosynthesis of certain amino acids, and to increased expression of acetohydroxyacid synthase, which is responsible for the conversion of pyruvic acid into its α-acetolactate diketones precursor (Brányik et al. 2008). Such changes are often associated with barriers to the mass transport of substrate and product, occurring in cases of encapsulation or entrapment. However, immobilization methods based on adhesion to solid surfaces have been described as less invasive in relation to the metabolic activity of immobilized microorganisms (Verbelen et al. 2006; Willaert 2000; Brányik et al. 2005,2008). For *S. cerevisiae* cells immobilized on DEAE-cellulose, used in the production of non-alcoholic beer, higher activity (in relation to free cells) of two glycolytic enzymes (hexokinase and PDC), was estimated (Van Iersel et al. 2000).

Many research studies have indicated an increase in the metabolic activity of immobilized cells that is manifested both by an increased rate of substrate use and by higher productivity of selected metabolites (Norton and D’Amore 1994; Angelova et al. 2000; Talebnia and Taherzadeh 2007; Nikoli et al. 2010; Bouallagui et al. 2013). In view of the volume of literature and data describing higher glucose flux (Junter et al. 2002; Plessas et al. 2007; Brányik et al. 2005) we would expect higher PDC activity in microorganisms immobilized than in suspended cells. In our study, we found higher PDC activity values only for *S. pastorianus* 680 during the fermentation with immobilized cells in comparison with free cells treated using the same process. However, in most cases, the experiments show that the immobilized microorganisms tested are characterized by a decrease in enzyme activity and ATP content. On the reduced metabolic activity of immobilized brewing yeast cells see also Masschelein et al. (1994).

The data obtained is very variable because it describes different type of yeasts. However, the our research complements not fully understood processes. The obtained results show that the activity of the immobilized cells may depend on the type, age or behavior of tested yeasts. Therefore, our study seems to be a valuable material for further studies on the area of physiology of immobilized yeasts.

## Conclusions

The reduced metabolic activity of immobilized yeast cells does not preclude benefits to their technological applications. A suitably large density of yeast cells per unit volume, possible only in immobilized cell systems, and a very short attenuation time (due to their resistance to environmental changes) produce a more efficient fermentation process. Several theories have been proposed to explain the enhanced fermentation capacity as a result of immobilization. Adhesion has a major influence on the plasma membrane properties of the yeast, which can cause modifications of some solute transport systems. The enhanced fermentation properties of immobilized cell systems could also be explained by the CO_2_ nucleation effect of the matrix (Verbelen et al. 2006). Moreover, a reduction in the ethanol concentration in the immediate microenvironment of the organism due to the specific adsorption of ethanol by the support may act to minimize end product inhibition. The greater volumetric productivity can be also a result of higher cell density in immobilized systems (Ivanova et al. 2011). However, our interesting results require a clear explanation in future studies.
